# Modification of TiAlV Alloys with Hybrid Layers Containing Metallic Nanoparticles Obtained by the Sol–Gel Method: Surface and Structural Properties

**DOI:** 10.3390/ijms23042283

**Published:** 2022-02-18

**Authors:** Magdalena Ziąbka, Katarzyna Matysiak, Katarzyna Walczak, Marcin Gajek, Katarzyna Cholewa-Kowalska

**Affiliations:** 1 Department of Ceramics and Refractories, Faculty of Materials Science and Ceramics, AGH University of Science and Technology, 30-059 Krakow, Poland; katarzyna.matysiak@poczta.onet.pl (K.M.); mgajek@agh.edu.pl (M.G.); 2 Department of Hydrogen Energy, Faculty of Energy and Fuels, AGH University of Science and Technology, 30-059 Krakow, Poland; walczakk@agh.edu.pl; 3 Department of Glass Technology and Amorphous Coatings, Faculty of Materials Science and Ceramics, AGH University of Science and Technology, 30-059 Krakow, Poland; cholewa@agh.edu.pl

**Keywords:** TiAlV alloy, silver, copper, zinc nanoparticles, composite hybrid layers, sol–gel, structural and surface properties

## Abstract

The aim of the work was to obtain hybrid coatings containing silver, copper, and zinc nanoparticles on the TiAlV medical alloy via a sol–gel process. The developed layers were designed to bring about a bactericidal and fungicidal effect, as well as for protection against surgical scratches during the implantation of implants used in veterinary medicine. In this work, the authors focused on evaluating the microstructure (SEM + EDS); the structure (XRD, FTIR); and the surface properties, such as wettability, free surface energy, and roughness of layers with various concentrations of metallic nanoparticles (2 and 5 mol %). Our results confirmed that the sol–gel method enables the easy manufacturing of hybrid layers endowed with different porosity values as well as various shapes and sizes of metallic nanoparticles. A higher concentration of nanoparticles was observed on the surface containing 5 mol % of metallic salts. The highest degree of homogeneity was obtained for the layers containing silver nanoparticles. In addition, the silver nanoparticles were round and had the smallest dimensions, even below 20 nm. The FTIR and XRD structural studies confirmed the presence of an organosilicon matrix containing all three types of the metallic particles. We conclude that the higher concentration of nanoparticles influenced the alloy surface parameters.

## 1. Introduction

Titanium and its alloys have been widely used in medicine for many years. Their applicability is based on the advantageous physical and chemical properties and good biocompatibility with human cells and tissues. Light, strong, and completely biocompatible titanium is one of the few materials that naturally meet the requirements of implantation in the human body. Titanium alloys have a much higher strength/mass ratio in comparison to stainless steel, their main competitor on the medical market.

The most frequently used titanium alloys are the ones with aluminum and vanadium (Ti6Al4V); with aluminum and niobium (Ti6Al7Nb); with aluminum and iron (Ti5Al2.5Fe); with niobium and zirconium (Ti13Nb13Zr); and with molybdenum, zirconium, and iron (Ti12Mo6Zr2Fe) [1,2,3,4]. Titanium and its alloys are most commonly used for orthopedic implants, such as hip joint prosthesis [5], knee implants [6], implants for external or internal stabilization of bone fractures [7], tiles, bolts, nails for osteosynthesis, spine stiffening devices [8], pins, bone tiles, bolts, intramedullary nails and external stabilizers [9,10], carbonized frames, and dental implants [11]. Titanium is also regularly used in the case of heart pacemakers and defibrillators, as a carrier structure for replacing the heart valve, and for intravascular stents [12]. Titanium is also used to develop external devices, as well as for orthotic terminals, artificial limbs, and surgical tools.

The Ti6Al4V alloy has optimal mechanical properties, very good corrosion resistance, and a two-phase structure α + β. The alloy is obtained by introducing an aluminum additive that reduces the specific gravity of titanium and strengthens a solid solution. Then, vanadium is introduced as a stabilizing element of the β phase [13]. However, the release of vanadium to the physiological environment is problematic, as it causes cytotoxic reactions and consequently neurogenic and carcinogenic disorders [14,15].

There are several approaches to reduce the penetration of ions in the environment and thus to increase the corrosion resistance and improve the tribological properties of titanium alloys. Firstly, the alloy elements may be carefully selected. Secondly, the alloys may be covered with protective layers. Thirdly, several techniques of surface processing may be applied. With regard to the layer formation mechanism, the modification techniques consist among others chemical and physical processes. Chemical methods include: chemical treatment, electrochemical treatment (anodic oxidation), sol–gel, chemical vapor deposition (CVD), and biochemical modification. In the case of physical methods, the formation of the surface layer on titanium alloys is mainly attributed to the thermal, kinetic, and electrical energy. These techniques comprise thermal spraying, physical vapor deposition (PVD), glow discharge plasma, and ion implantation [16,17,18].

The mentioned above, sol-gel is a chemical method used to synthesize ceramic or glass materials in a relatively low temperature [19] through hydrolysis and condensation of metal alkoxide precursors. One of its advantages is the low heating temperature that does not result in thermal stresses in the material. This method is therefore used to obtain bioactive layers (e.g., hydroxyapatite, bioglass) that protect the metallic implant surface, improving the implant–bone integration via better adhesion and cell proliferation [20]. In addition, this technique is low-cost, and it allows for the coating of elements of geometrically complex shapes [21]. On the other hand, the sol–gel method results in a fragile inorganic component that can be eliminated by introducing organically modified precursors. This method allows for the creation of flexible hybrid organic–inorganic materials with the possibility of tailoring and modifying their composition, structure, and thickness to the desired application [22,23].

An important problem appearing when using medical implants is a bacterial infection leading to chronic infections and prolonged antibiotic therapy. In some cases, it is even necessary to remove the implant, triggering another operation and additional stress and pain for a patient, leading to longer hospitalization. It is also connected with higher costs of treatment and rehabilitation. In many cases, an issue is the bacterial resistance to an antibiotic therapy. In the sol–gel method, the bactericidal effect can be ensured, inter alia, by introducing antibacterial agents such as silver, copper, and zinc [24]. The sol–gel technique offers a unique opportunity to incorporate antibacterial components into a pure organically modified silica matrix. By dissolving Ag/Cu/Zn nitrates in the silica sol, one can obtain a metal-doped coating on various surfaces by the simple coating technique, such as dipping, spraying, or spinning [25,26,27,28]. When designing hybrid layers with biocidal properties, one should also remember that the process of developing materials itself is extremely important. Both the precursors and the synthesis conditions have to be precisely selected in order to ensure adequate physicochemical and biological properties. These factors are crucial in order to obtain homogeneous and resistant layers that meet the mechanical and biological requirements, such as biocompatibility and bactericidal activity [29].

The presented study describes an attempt to develop the homogeneous organic–inorganic layers containing nanoparticles of biocidal metals (silver, copper, zinc) on the substrate of the Ti6Al4V alloy. The sol–gel technique was used to protect the modified substrates against corrosion and scratching protection and to ensure antibacterial action while maintaining the biocompatibility of materials. This article is the first part of the research investigating the characteristics of the microstructural and surface properties of the obtained layers. In the next work, we will describe the bactericidal properties and confirm the biocompatibility of the developed layers.

In this work, scanning electron microscopy (SEM), X-ray diffractometry (XRD), Fourier transform infrared (FTIR) spectroscopy, profilometry, wettability, and confocal microscopy were employed to analyze the surface chemical compositions, microstructure, phases, and surface properties.

## 2. Results and Discussion

**Figure 1:**
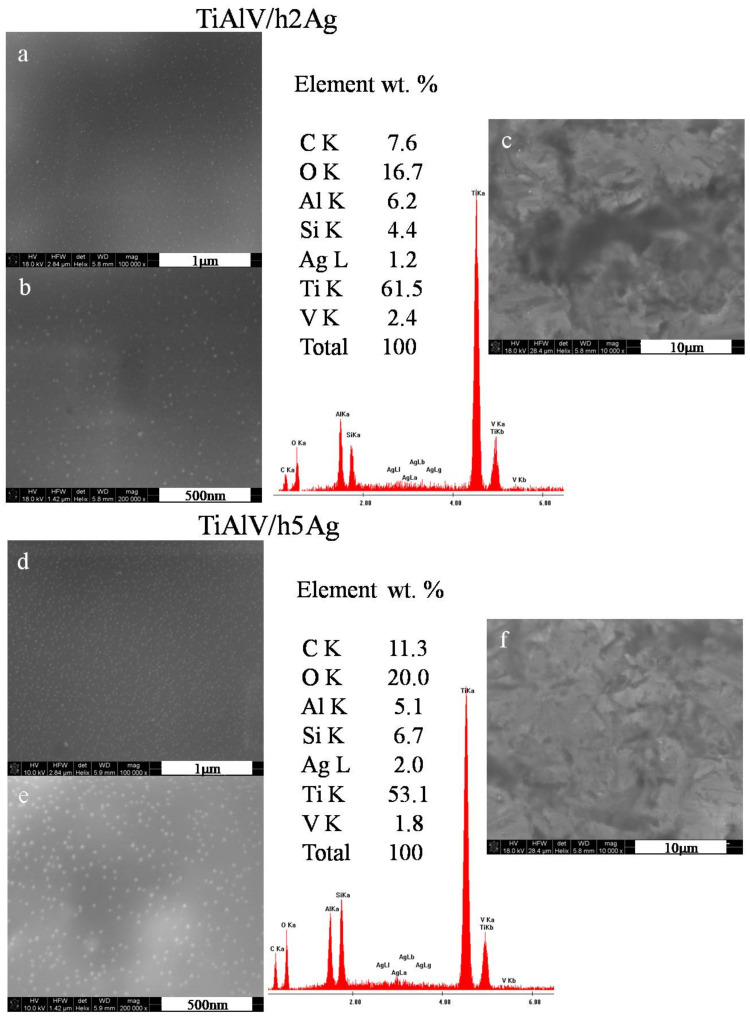
SEM images and EDS spectra of titanium alloy (TiAlV)-covered sol–gel hybrid layer containing silver nanoparticles; (**a**–**c**) containing 2% AgNPs, and (**d**–**f**) containing 5% AgNPs.

The organic–inorganic sol–gel materials with increasing amounts of Ag/Cu/Zn were successfully synthesized and applied as coatings onto the TiAl4V discs. As anticipated, NPs formed as a result of chemical reactions in sols, and subsequent thermal treatment of coating was observed in the layers. The SEM surface observations confirmed the presence of metallic nanoparticles on the surface of the TiAlV alloy. In the case of layers containing silver nanoparticles, a homogeneous distribution of spherical nanoparticles was revealed over the entire observed surface (Figure 1a,b). The silver particles size was estimated on the basis of 10 measurements made with the automatic function of measuring the particle diameter with a microscope ruler. The AgNP particle diameter was 5–20 nm for the layers containing both 2 and 5 mol % of the silver nitrate precursor. The average EDS chemical analysis performed in the area of 285 × 285 µm confirmed the presence of silver in the layers. The silver content constituted about 1% by weight for the layers with 2 mol % of the precursor. Observations of the silver-doped layers at a magnification of 10,000× showed that the layers were homogeneous, and no porosity or presence of agglomerates was observed (Figure 1c,f). In the case of layers containing 5 mol % of the precursor, a higher proportion of silver nanoparticles was observed (Figure 1e). This was also confirmed by the EDS analysis, which showed the 2% weight of AgNPs for the layers with 5 mol % of the precursor.

**Figure 2:**
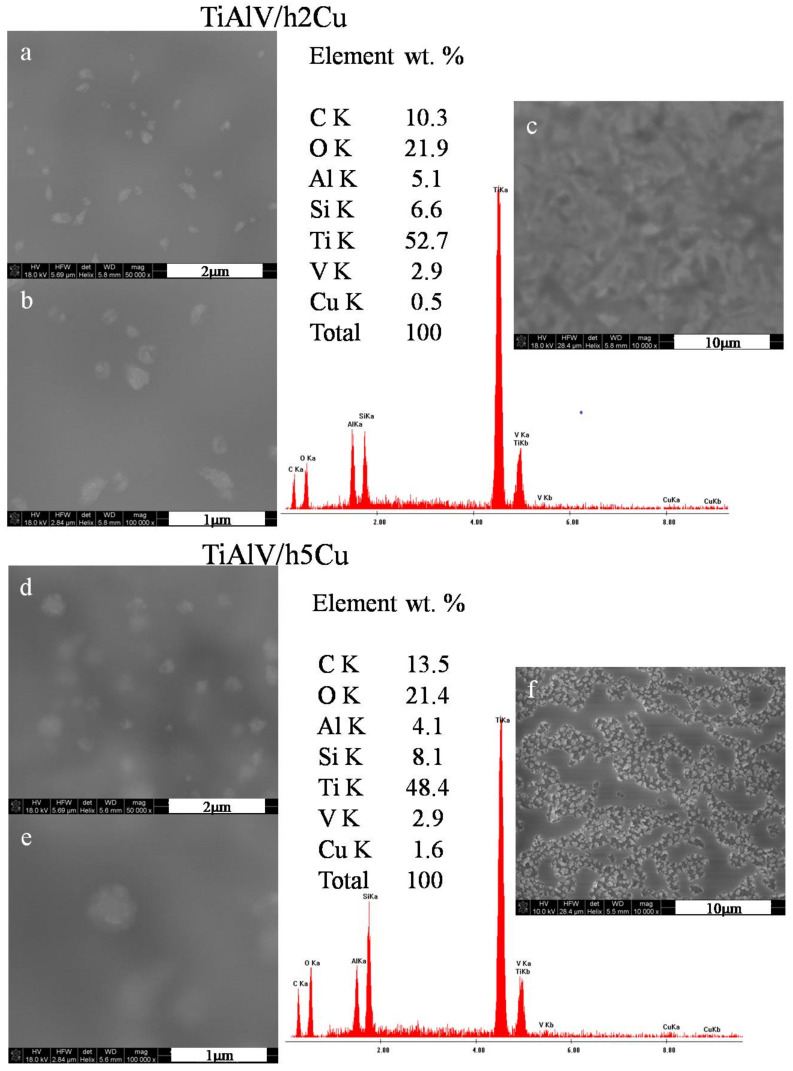
SEM images and EDS spectra of titanium alloy (TiAlV)-covered sol–gel hybrid layer containing copper nanoparticles; (**a**–**c**) containing 2% CuNPs, and (**d**–**f**) containing 5% CuNPs.

The surface observations of the layers containing copper nanoparticles revealed the presence of particles measuring 100–500 nm in diameter (Figure 2a,b,d,e). The particles were not distributed homogeneously—there were areas with no particles at all, as well as areas with spherical agglomerates of 500 nm (Figure 2e). The particles with the appearance of flocculent clouds dominated on the layers surface. In the case of layers containing 2 mol % of copper nitrate precursor, the layer appeared to be smooth and uniform, with no signs of microcracking or porosity (Figure 2c). On the other hand, for the layers containing 5 mol % of copper precursor, both an increase in the content of copper particles and an increase in their diameter was observed. The areas with high concentration of nanoparticles were visible at the magnification of 10,000× (Figure 2f). The average EDS analysis performed in the area of the layers 285 × 285 µm revealed a copper concentration of 0.5 wt % for the TiAlV/h2Cu layer and 1.6 wt % for the TiAlV/h5Cu layer.

**Figure 3:**
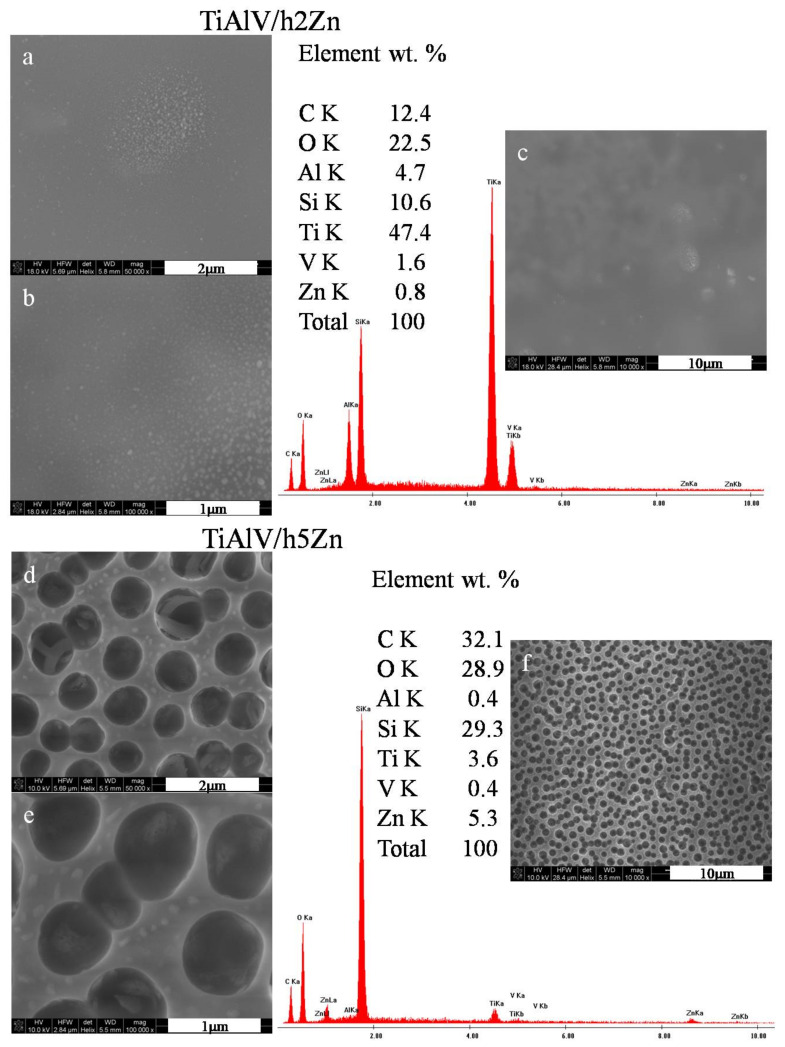
SEM images and EDS spectra of titanium alloy (TiAlV)-covered sol–gel hybrid layer containing zinc nanoparticles; (**a**–**c**) containing 2% ZnNPs, and (**d**–**f**) containing 5% ZnNPs.

The microstructural observations of the zinc-doped layers revealed the presence of zinc nanoparticles measuring 20–85 nm in diameters for the layers with 2 mol % zinc nitrate precursor (Figure 3a,b) and 60–140 nm for the layers with 5 mol % precursor. There were also the areas with local concentration of zinc nanoparticles observed. At 10,000× magnification (Figure 3c), no porosity was noticed, and the surface of the layer was smooth. On the other hand, for the TiAlV/h5Zn layers, the network porosity with round pores appeared, with a pore size of about 700–900 nm (Figure 3d–f). The average EDS analysis performed for the areas analogous to the silver and copper layers confirmed the chemical presence of zinc of 0.8% by weight for the TiAlV/h2Zn layer and 5.3% by weight for the TiAlV/h5Zn layer. The ZnNPs nanoparticles were present both in the pores and on the network surface.

The films, prepared with AgNO_3_, regardless of the concentration, exhibited AgNPs 5–20 nm in diameter, and only the number of Ag clusters increased with the increasing AgNO_3_ content. In the case of the Cu- and Zn-doped coatings, the nanoparticle size and number increased with the increasing initial components concentration. The formation of the Ag/Cu/Zn particles suggested that a reduction of the ionic form to colloidal metal had occurred. It was reported that the functional groups (amine, thiocyanate) as well as the organically modified silanes acted as a complexing and reducing agent for metal ions in sol–gel-derived SiO_2_ coatings. In this study, we also expected that the organic groups of the used organically modified silane (TMSPM) would act as a redactor and lead to the metal colloid formation during the proposed thermal treatment in air [30,31,32,33].

**Figure 4:**
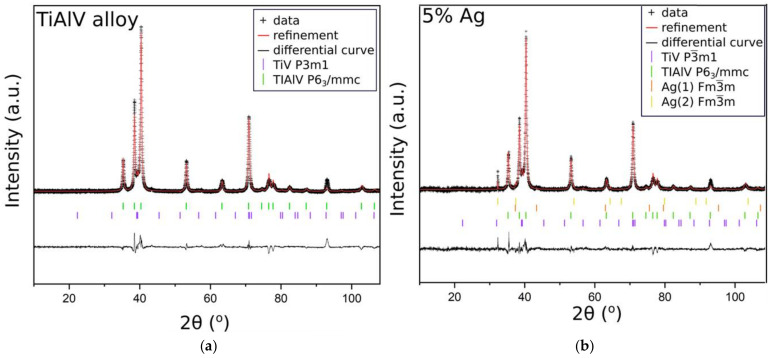
XRD patterns of the TiAlV alloy (**a**); the TiAlV alloy with 5% silver nanoparticles in the SiO_2_ matrix (**b**).

In order to confirm that the base sample contained TiAlV alloy, we employed the XRD measurements. The diffractogram in Figure 4a indicated the presence of high peaks, originating from hexagonal (*P6_3_/mmc*) and trigonal (*P* $\bar{3}$ *m1*) space groups, which were ascribed to metallic alloys TiV and TiAlV, respectively. These results stayed in line with our previous studies [34]. Figure 4b shows the diffractogram of the base sample with the antibacterial layer consisting of the SiO_2_ matrix with silver nanoparticles (5%). Since the peaks from TiV and TiAlV were of high intensity, it was only possible to see small reflections coming from silver, especially at around 32.2°, 37.1°, and 43.4°.

**Figure 5:**
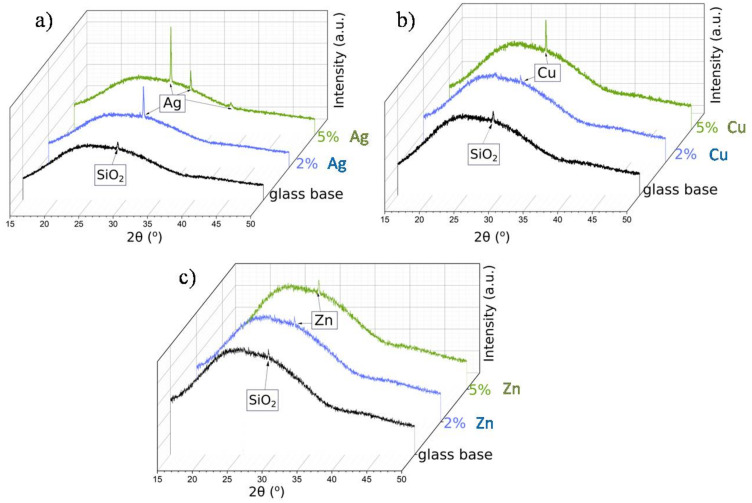
XRD patterns of the metallic nanoparticles in SiO_2_ matrix on the glassy base: Ag (**a**), Cu (**b**), and Zn (**c**) at various percentage contents.

It must be noted that for a smaller content of metallic nanoparticles, similar observations were not possible. Thus, in order to confirm the presence of metallic nanoparticles in other samples, we needed to conduct the measurements at the glassy (amorphous) base (Figure 5). For all samples, the broad hump (at 2θ: 25–35°) typical for the amorphous phase was observed. Moreover, the diffractogram of the glass base revealed the presence of a small peak, near 29° 2θ angle, assigned to crystalline SiO_2_ [35]. The XRD patterns of the samples with silver, copper, and zinc nanoparticles showed that the main peak overlapped with the SiO_2_ reflection. What is more, this reflection increased with the increase in the metallic nanoparticles content in the matrix. Nevertheless, despite the glassy base, the visible reflections were small and low-intensity—a result of the grain nanosize (see the SEM analysis).

**Figure 6:**
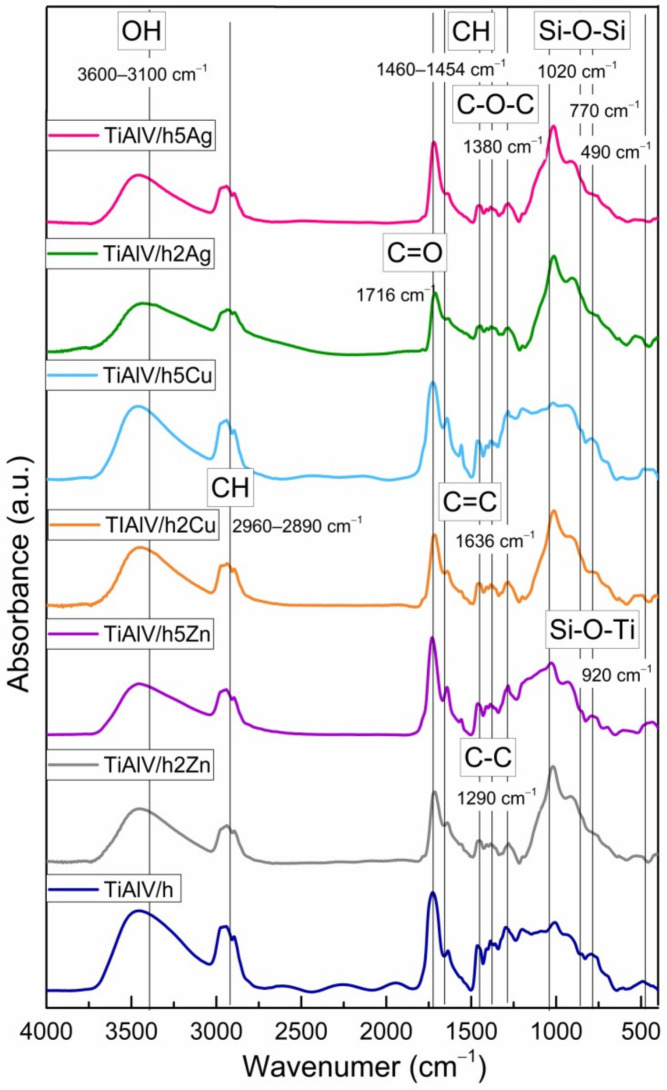
FTIR spectra of titanium alloy (TiAlV)-covered sol–gel hybrid layer containing 2 and 5 mol % silver, copper, and zinc nanoparticles.

Figure 6 shows the FTIR spectra of the coatings in the 400–4000 cm^−1^ region. In all the spectra, the wide band located in the 3100–3600 cm^−1^ spectral region was assigned to the contribution of physically and/or chemically incorporated water, corresponding to the OH symmetric stretching from asymmetrically hydrogen bonded water. The CH alkyl stretching bands were observed in the 2890–2960 cm^−1^ region [36,37]. The peaks at 1636 and 1716 cm^−1^ were characteristic for the C=C and C=O vibrations in the methacrylate group of TMSPM, respectively. Additionally, the vibrations bands at about 1454–1460, 1380, and 1290 cm^−1^ were assigned to the C-H bending vibration, the C-O-C skeletal vibrations, and the C-C stretching vibrations, respectively [38,39]. It is likely that the band at 1380 cm^−1^ also covered the CH_3_ rocking vibrations. The Si-O-Si structure formation was supported by the presence of the strong asymmetric Si-O-Si stretching absorption at about 1020 cm^−1^, while the peak at 920 cm^−1^ reflected the contribution of the Si-O-Ti bond in the material structure, confirming the reaction of TIP with silane compounds, which leads to the cross-linked structure with incorporated Ti atoms [40,41]. The presence of additional peaks of the Si-containing groups at 770 and 490 cm^−1^, which were, respectively, assigned to the symmetric stretching and bending vibration modes of Si-O-Si bonds, also indicated the spatial silica-based structure formation.

In addition, the slight shift of the carbonyl absorption band to the frequency lower than 1720 cm^−1^, characteristic of carbonyl groups, revealed the formation of hydrogen bonds with the silane (TEOS) hydroxyl groups as well as the fact that the interaction between the carbonyl groups and metal nanoparticles probably occurred mainly through a physical force.

The FTIR data revealing the presence of bands associated with organic groups and the appearance of Si-O-Si and Si-O-Ti vibrations confirmed the copolymerisation of respective compounds, resulting in the formation of a homogeneous organic–inorganic hybrid layer.

**Figure 7:**
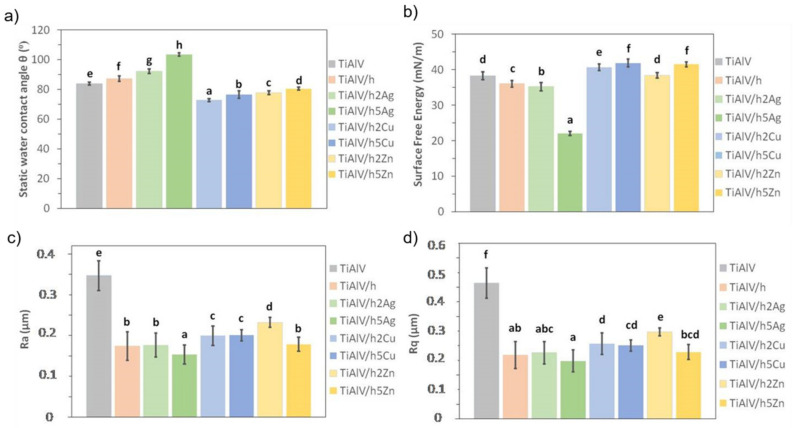
Static water contact angle (**a**), surface free energy (**b**), and roughness parameters (**c**,**d**) of titanium alloy (TiAlV)-covered sol–gel hybrid layer containing silver, copper, and zinc nanoparticles. Statistically significant differences (*p* < 0.05) in the studied parameters between the different coating types are indicated by different lower-case letters. Bars marked with the same letters are not statistically different.

The TiAlV alloy modification with organic–inorganic layers had a significant impact on their surface properties. Covering the alloy with the base layer (TiAlV/h) resulted in an average increase in the contact angle by 0.5° and a decrease in the surface energy by 0.2 mN/m (Figure 7a,b). In the case of the silver-doped layers, an increase in the value of the contact angle was observed, along with a higher nanoparticle content. For the 2% AgNPs layers, the contact angle increased by an average of 6°, and for the 5% AgNPs, the value increased by an average of 17°. Thus, a clear change from hydrophilic to hydrophobic nature was observed in the layers, which correlated with the surface energy change. The highest AgNPs caused an energy decrease by an average of 14.6 mN/m, as compared to the pure alloy.

In the case of copper and zinc layers, a significant decrease in the contact angle and an increase in the surface energy were observed. The values of both parameters correlated with the content of metal nanoparticles. The smaller the proportion of metallic nanoparticles, the lower the contact angle and the higher the surface energy. For the 2% CuNP layers, the reduction of the contact angle was on average equal to 9°, and the energy increase was on average 0.3 mN/m. For the 5% CuNPs layers, the contact angle decrease was 4° on average, and the average energy increase was 1.3 mN/m. As for the surface energy, for the 2% ZnNPs layers, the decrease in the contact angle was on average 3.8°, and there were no changes in the surface energy value. For the layers containing 5% ZnNPs, the contact angle decreased by 1.5° on average, and the energy value increased by 1.4 mN/m on average.

When analyzing the surface roughness, we observed that the modification of the TiAlV alloy with the sol–gel layers significantly improved the samples’ surface smoothness. The decrease in the Ra and Rq parameters was observed for all the tested layers in relation to the base alloy. In the case of the pure hybrid layer, the Ra parameter decreased by 0.10 µm on average, and the Rq parameter by 0.15 µm. The introduction of metallic particles into the layers also reduced roughness parameters in comparison to pure alloy. Yet, only in the case of the 5 mol % Ag layers was the even lower Ra parameter value (reduction by 0.04 µm) recorded, as compared to the base layer. The addition of copper and zinc nanoparticles increased the Ra parameter, as compared to the base layer. Similar behavior was observed for the Rq parameter. The increase in the roughness of the layers containing copper and zinc nanoparticles related to the size of the particles formed in the layer their uneven distribution and the apparent porosity. Thus, the highest surface smoothness was obtained for the layers containing silver nanoparticles, which correlated with the SEM observations.

**Figure 8:**
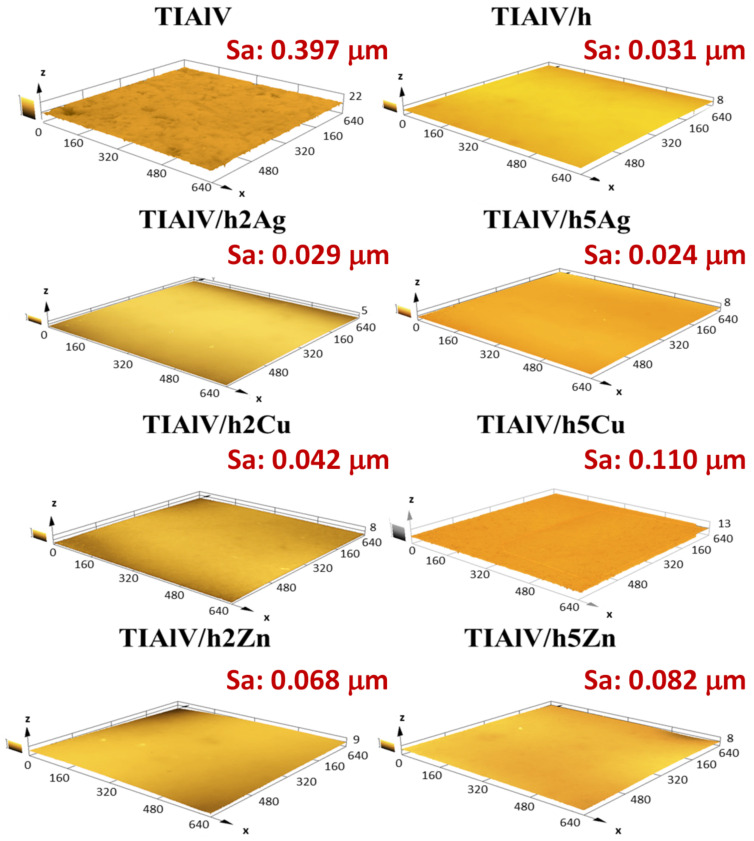
Three-dimensional confocal microscope microphotographs with area roughness parameter (Sa) of titanium alloy (TiAlV)-covered sol–gel hybrid layer containing silver, copper, and zinc nanoparticles. The scale in the charts is described in μm.

The surface roughness examinations performed with a confocal microscope (Figure 8) confirmed the convergence with the results obtained from the profilometer. The analogous values of the Sa parameter were observed for all the tested samples. The modification of the TiAlV alloy surface with the hybrid layers increased the surface smoothness. A significant decrease in the Sa parameter was observed for all the layers, while the highest surface smoothness was obtained for the 5% AgNPs layer, with the Sa parameter 0.373 µm lower than the pure alloy. The attempts to estimate the wetting angle of the coatings containing AgNPs, CuNPs, and ZnNPs showed that the influence of the surface topography was predominant. Such a significant increase in the contact angle for the 5 Ag layer took place most likely due to the high concentration and the uniform distribution of nanoparticles. It was reported that the WCA (water contact angle) increased with the silica nanoparticle concentration increase; however, in that study, the contact angle increase may have been attributed to the surface roughness increase, contrary to our results [42]. As for the films with CuNPs and ZnNPs, it was most probably the case that the creation of porosity (SEM observation) made the samples more hydrophilic than the Ag-doped layers, due to the capillary lift of liquids through this network of pores [43,44].

## 3. Materials and Methods

### 3.1. Material Manufacturing

Metallic substrates (disks with a diameter of 1 cm) made of the TiAlV alloy were manufactured and delivered by Medgal (Księżno, Poland). Composite hybrid (organic-inorganic silicate) sols for coating their surface were prepared by the sol–gel method. The chemical compositions of layers were designed with 2 and 5 mol % concentrations of metallic precursors, respectively. The coating solution was prepared using the following precursors: TEOS (tetrethylorthosilicate, Si(OC_2_H_5_)_4_); TMSPM (3-(Trimethoxysilyl) propyl methacrylate, H_2_C=C(CH_3_)CO_2_(CH_2_)_3_Si(OCH_3_)_3_); TIP (titanium (IV) isopropoxideTi(OC_3_H_7_)_4_); and nitrates AgNO_3_, Cu(NO_3_)_2_×3H_2_O, and Zn(NO_3_)_2_×6H_2_O (Sigma Aldrich, Krakow, Poland) as a source of metallic ions.

First, the metallic precursor was dissolved in propanol, serving as a solvent. The solution was mixed for 30 min in a magnetic stirrer. Next, TEOS, TMSPM, and TIP were successively added to the solution. Having introduced each of the reagents, we then stirred the solution for 20 min. The aqueous solution of the HNO_3_ nitric acid was used as a reaction catalyst. The volume ratio of all the components to the propanol solvent was 1:8. Prior to layering, the solution aging time was 24 h, and the viscosity equaled 6 cP. The obtained sol was stored in a sealed container, with no access to UV and VIS radiation in order to limit the Ag^+^ reduction. The discs were washed with propanol. The layers were applied by the dip coating technique with the 50 mm/min withdrawal speed, and then the coated discs were drying in ambient conditions for 24 h. Next, the applied layers were stabilized by the two-step thermal treatment at 80 °C for 10 min and 130 °C for 15 min.

The following nomenclature was adopted to standardize the names of the samples in this work (Table 1).

### 3.2. Material Examinations

#### 3.2.1. Scanning Electron Microscopy

The detailed microstructure examination of the materials was carried out by means of the Nova NanoSEM 200 scanning electron microscope (FEI, Eindhoven, The Netherlands) with the Genesis XM X-ray microanalysis system (EDAX, Tilburg, The Netherlands) featuring the EDAX Sapphire Si(Li) EDS detector. The observations and measurements took place in low vacuum conditions using a Helix detector (SE) with the accelerated voltage of 10–18 kV.

#### 3.2.2. XRD X-ray Diffractometry

The XRD patterns of the samples were obtained using Malvern PANalytical Empyrean diffractometer (Worcestershire, UK) equipped with a copper lamp (monochromatic radiation with 0.1530598 nm CuKα1 and 0.1544938 nm CuKα2 lines) and a PiXcel3D detector. All the samples were measured in Programmable Divergence Slit (PDS) mode in two ways: (1) silver, copper, and zinc nanoparticles in the SiO_2_ matrix were spread on the metallic (TiAlV) alloy; (2) silver, copper, and zinc nanoparticles in the SiO_2_ matrix were spread on the glass plate.

The data analysis was carried out using X’pert High Score software with PDF + 2021 database, and it was supported by the Rietveld analysis using GSAS + EXPGUI software [45,46].

#### 3.2.3. Fourier Transform Infrared Spectroscopy, FTIR

The structural changes caused by the chemical modification of the substrates with the sol–gel layers were investigated using the BTS-RAD FTS 3000 Excalibur spectrophotometer (Bio-Rad, Hertfordshire, UK). The ATR technique (MIRACLE attachment equipped with ZnSe doped diamond crystal) was used. The tests were carried out in the range of 400–4000 cm^−1^ with a resolution of 4 cm^−1^.

#### 3.2.4. Surface Wettability

The static water contact angle (WCA) was used to assess the surface wettability via the sessile drop method with the automatic drop shape analysis system DSA 10 Mk2 (Kruss GmbH, Hamburg, Germany). The constant temperature and humidity conditions were maintained throughout the tests, while the UHQ-water droplets of 0.25 μL were applied on each pure and dry sample. We calculated the apparent contact angle as an average of 30 measurements and expressed it as the mean ± standard deviation (SD).

#### 3.2.5. Surface Free Energy

The free surface energy was determined by testing the contact angles of two measuring liquids: ultra-pure distilled water (UHQ PURE Lab., Vivendi Water, Paris, France) and diiodomethane. The measurements were carried out on the DSA 10 Mk2 optical apparatus (Kruss GmbH, Hamburg, Germany) at room temperature. The test pattern was identical to the contact angle test. The obtained photos of drops of two measuring liquids established the free surface energy on the basis of the values of the dispersion and polar components. The value was the arithmetic average obtained from 30 measurements. The results are presented with standard deviations (SD).

#### 3.2.6. Roughness

The arithmetical mean roughness (*Ra*) and the root mean square roughness (*Rq*) of the investigated materials were evaluated by means of the contact profilometer HOMMEL-ETAMIC T1000 wave (Jenoptik AG, Jena, Germany). The arithmetical mean roughness values were an average of 10 measurements with traverse length 4.8 mm according to the EN ISO 4288 expressed as the mean ± standard deviation (SD).

#### 3.2.7. Confocal Microscopy

The surface topography of samples was observed using the laser confocal microscope Lext OLS 4000 (Olympus, Tokyo, Japan) with the magnification 50×. The scanned area was 640 × 640 μm.

#### 3.2.8. Statistical Analysis

The results were analyzed using one-way analysis of variance (ANOVA) with Duncan’s posthoc tests, which were performed with Statistica 13.1 software (TIBCO Software Inc., Palo Alto, CA, USA). The results were considered statistically significant when *p* < 0.05.

## 4. Conclusions

The conducted microscopic studies proved that it was possible to obtain hybrid layers with the different microstructures of the layers themselves and various nanoparticle morphologies. The layers obtained by the sol–gel technique differed depending on the precursor used. The conducted research confirmed that silver, copper, and zinc nanoparticles differed in shape and size. The studies showed that silver nanoparticles were the most evenly distributed in the organosilicon matrix, while copper and zinc nanoparticles formed agglomerates, and zinc caused layer porosity. The FTIR and XRD structural studies confirmed the silicon matrix and metal nanoparticle presence, respectively. The TiAlV alloy layer modification changed the surface properties, such as wettability, surface energy, and roughness. The layers containing silver nanoparticles increased the value of the contact angle, thus lowering the surface energy. The layers containing copper and zinc nanoparticles showed different behavior. The modification of the titanium alloy with hybrid layers certainly affected the roughness parameter, causing its significant decrease. Thus, the performed tests proved that it was possible to smooth the alloy surface and protect it against surgical scratches and scrapes during the same process.

## Figures and Tables

**Table 1:** Samples nomenclature.

Sample Characteristic	Sample Nomenclature
Titanium alloy Ti-6Al-4V	TiAlV
Titanium alloy Ti-6Al-4V with pure hybrid layer	TiAlV/h
Titanium alloy Ti-6Al-4V with hybrid layer containing 2 mol % AgNPs	TiAlV/h2Ag
Titanium alloy Ti-6Al-4V with hybrid layer containing 5 mol % AgNPs	TiAlV/h5Ag
Titanium alloy Ti-6Al-4V with hybrid layer containing 2 mol % CuNPs	TiAlV/h2Cu
Titanium alloy Ti-6Al-4V with hybrid layer containing 5 mol % CuNPs	TiAlV/h5Cu
Titanium alloy Ti-6Al-4V with hybrid layer containing 2 mol % ZnNPs	TiAlV/h2Zn
Titanium alloy Ti-6Al-4V with hybrid layer containing 5 mol % ZnNPs	TiAlV/h5Zn

## Data Availability

Not applicable.
